# Synopsis of Contributions to the Physiology of Digestion

**Published:** 1859-07

**Authors:** W. Busch, Louis Bauer

**Affiliations:** Bonn.; Surg. to the L. I. College Hospital, Brooklyn, &c.


### ARTICLE XIII.

*Synopsis of Contributions to the Physiology of Digestion.* By Prof. W. Busch, of Bonn. Collaborated by Louis Bauer, M. D., M. R. C. S., Eng., Surg. to the L. I. College Hospital, Brooklyn, &c.

With the aid of experiment, the physiology of digestion has been elaborated beyond anticipation; yet doubts are still entertained whether experiments upon the higher mammalia permit conclusive inferences to be drawn upon the digestive functions in man. For this reason the contributions of the talented professor of surgery at the University of Bonn, as contained in the recent number of Virchow's Archives of Pathological Anatomy and Physiology, (vol. xiv., No. 2,) will be received with great satisfaction, having had the rare opportunity of testing upon human digestion the various views on that process hithero entertained.

A woman, thirty-one years of age, was introduced to the professor, who, from injuries received, presented fistulous openings, which completely separated the superior portion of the intestinal track (stomach, duodenum, and a short fragment of the jejunum) from the lower one.

As not the least communication existed between the two portions, and as the alimentary contents of the stomach and duodenum, conjointly with the gastric, enteric, and pancreatic succus and bile, were discharged separately, from the contents and secretions of the lower portion, a series of experiments could thus be instituted that were well calculated to throw light upon some obscure points in the physiology of digestion, whilst it affirms others, thus converting hypothesis into established facts.

The woman in question was in her sixth month with her fourth child, when she was assailed by a bull. One horn lacerated her abdominal parietes, midway between the pubic symphysis and umbilicus, whilst the other caused but a trifling ecchymosis. The wound (in the parietes) was five inches in length, and transversely to the mesian line of the body. The prolapsed intestines seemed not to be injured. They were replaced, and the wound closed by sutures.

During the subsequent three days the patient passed one alvine evacuation, and felt comparatively comfortable; but then violent pains commenced, the wound reopened, stools became obstructed, and with increased pain and suffering, food and fæces forced their way through the opening. A week after, the patient was prematurely confined with a still-born child.

Although the pains very soon after entirely subsided, and although a most ravenous appetite set in, yet the patient became so rapidly reduced in flesh and strength, that her relatives decided upon her removal to Bonn. It appears from collateral statement, that the intestinal track had not been injured at first, but had subsequently become inflamed and sloughed.

When Professor Busch first saw the patient, she had the appearance of a woman double her age, presenting a rare degree of emaciation, without the slightest trace of fat. The epiphyses of the bones were everywhere prominent, and the muscles attached flabby, relaxed, and seemingly atrophied. It hardly needs mentioning, that the patient was equally debilitated and prostrate; that she had scarcely strength enough to move her lips, and that her expression of countenance was that of profound indifference and indolence. Pulse rarely exceeded 50, and was of small, empty, thready calibre ; respiration correspondingly rare (10 to 12 in a minute) and superficial, with a weak, hoarse, and toneless voice.

On examining the abdominal parietes, which were drawn towards the spine and excavated, an oval distension presented itself, about midway between the pubic symphysis and umbilicus, similar to abdominal hernia. On the lower half of said projection there was a cutaneous defect of about one and a half inches, with two openings, a left and superior and a right and inferior one. The finger, or a middle-sized elastic tube, could easily enter the former, from which a yellowish liquid issued, occasionally mixed with fragments of food. The latter was surrounded by a prolapsed ring of mucous membrane, which also admitted the finger, but for only a short distance, where a valve or stricture stayed its progress. Dilatation soon overcame the difficulty, so that no further interruption existed between that opening and the anus.

From this pathological condition, it follows that there was a perfect artificial anus of the intestine, which had no communication whatsoever with the lower portion of the track.

The seat of the superior fistula close to the umbilicus, the density of Kerkring's connivent valves, and the liquid state of chyme with unaltered fragments of food, rendered it highly probable that it connected not only with the smaller intestine, but that its connection could not be far from the diverticulo Vateri. This supposition was still more strengthened by the fact, that in the morning, and prior to breakfast, the bile discharged was green as grass, whilst that color was not met with in a lower portion of the bowels ; and still more by the rapid waste of the patient, despite of rapacity for, and abundance of, aliments consumed.

Unfortunately, the patient's weight was not ascertained when admitted to the University Hospital, but her emaciation may be imagined by comparison, for after considerable improvement of nutrition, she weighed, eight weeks after, no more than 68 pounds, 2 ounces; whereas, thirteen weeks after, she weighed 75 pounds; and twenty-one weeks after, 85 pounds.

An operation, purposing to connect the two fragments of the intestinal tube, was of course, out of the question at that juncture, and the whole attention was directed upon leading the contents of the upper portion into the lower by artificial means. But failing repeatedly in that attempt with various appliances, another course of feeding the patient was determined on and followed up successfully. At first, protean substances were injected into the lower opening, alternately with amylaceous, and subsequently, eggs and meats were stuffed in by the finger. The result was most surprising, and admitted no comparison with the previously adopted feeding through the mouth. Although there was not commensurate increase of the volume of the patient, yet the muscles manifested more tone, the features lost their death-like expression, the eyes became bright, the voice returned, and the patient could sit up in the erect posture.

After the patient had acquired a certain degree of strength and weight, the food was taken through the mouth, which almost sufficed to maintain nutrition, needing but rare additional support through the lower portion of the intestine.

Professor Busch had thus ample time and scope for his digestive experiments and observation, which the following will illustrate:

1. *Consequences of Wear and Tear with Deficient Assimilation.*

When the patient entered the hospital, the immediate effects of the injuries received had passed off, and her morbid appearances were solely attributable to the immense loss of digestive fluids, amounting to several pounds a day, without adequate supply. Bidder and Schmidt observed the same effects in dogs, with artificial fistulæ of the small intestine. The disappearance of fat, the sequent loss of muscular strength, somnolence and decrease of temperature, are well known facts. The latter is, however, but subjective, and not confirmed by the thermometer. With the progressive improvement of the patient, she became more comfortable and wakeful. When her prostration was greatest, her time was actually divided between eating and sleeping; but at a later period she required no more sleep than a healthy individual. Hoarseness and voicelessness had no other cause than inanition; for when, at a later period, the closure of the fistula was attempted, and the patient voluntarily abstained from taking any food for several days, in order not to disturb the healing process, both returned to a certain extent for the time being. Vox cholerica may be ascribed to an analogous cause.

Highly interesting were the statements of the patient as to sensation of hunger. She devoured incredible quantities of food, and for a length of time, while still eating, the food first taken would make its appearance in the superior fistula, and on being questioned, she would state that, although feeling better, her strong desire for food was not satiated. In fact, though her stomach might be filled, she nevertheless felt an irresistible desire for aliments, being of course materially increased by abstinence.

The case was decidedly most favorable as to the physiological character of hunger, the two factors of which were clearly discernible, viz. that, general sensation, originating with the extreme want of supply, and that appertaining to the emptiness of the first passages themselves. The latter could be temporarily satisfied; the former did not subside before the supply had somewhat restored the balance with the continuous waste and drainage. Another proof of the dualism of hunger was rendered by the observation that the stuffing of the lower section of the intestinal tube reduced the hungry feeling more effectually and permanently than the continuous reception of food by the stomach.

### 2. *Peristaltic Motion.*

The entire absence of adipose tissue, and the extreme attenuation of the skin in general, would have enabled the observer to watch and to follow the peristaltic movements of the bowels. But this case was still more favorable for such observations, by the co-existence of abdominal hernia and the direct exposure of a portion of the entrails.

Under ordinary circumstances the peristaltic motions were uniform in all parts of the bowels; but when, as it occasionally happened, a prolapsed invagination took place through the superior fistula, the invaginated portion would move vehemently until retracted, and sometimes tonic spasms would befall it so as to be hard and erect. This, however, only happened when the prolapse measured more than two inches.

According to Ludwig's and Schwarzenberg's observations, the peristaltic motions are periodical, alternating with perfect rest of the intestines, and this was found to be correct in the case of Professor Busch. The latter saw, oftentimes, during 10–15 minutes, not the slightest alteration in the calibre of the bowels. Nor could he find that the difference of temperature, or a moderate mechanical stimulus (for instance, the introduction of a finger) made any difference. Nay, even food did not always disturb the quietude. The same would, however, suffice to increase or rather accelerate the peristaltic actions. Moreover, the latter could be observed early in the morning, before the patient had taken food, showing a certain independence of external influences. During night rest seemed to supervene, when also the discharge ceased from the superior section, commencing again at 4 or 5 o'clock, A. M. Light food, taken late, would entirely escape before night; heavier victuals would eliminate in part, and the rest in the morning. Sleep or waking made no difference at any time. The respective contents of the intestines are not propelled in a continuous column, but with interruption. Substantial food would be pressed out like part of a soft sausage; liquids would issue briskly forth, and then a pause would ensue, and so on. If not attributable to the adhesion of the bowels to the abdominal parietes, gotten up for the purpose of freeing itself, and consequently connected with the morbid condition of the patient, there is also a longitudinal locomotion of the intestinal track. For the peristaltic movement is not exclusively circular, but also longitudinal, and has been clearly observed so in the present instance. A finger being introduced in the intestinal tube, the latter would close around it, and cause a sensation as if the finger was drawn deeper in. The intestine would also glide over the finger, and then contract to expel the foreign body. In the expulsion of food and digestive liquids, the same could be observed in the periodical protrusion and retraction of the mucous membrane through the superior fistulous opening.

The experiments of Ludwig and Schwarzenberg upon dogs, as to the direction of the peristaltic motion, tending to show that it is invariably towards the anus, could not be confirmed in Prof. Busch's case, the lower section of the intestinal tube being repeatedly observed to move antiperiastically, and disturb thereby the experiments in progress. Thus not only food, hours and days after being taken, was returned from the inferior opening, but also a gauze bag filled with articles of food, and deeply introduced. Soon after the dilatation of the lower fistula, mucus was ordinarily thrown out by regurgitation.

The various experiments instituted, with a view to ascertain the exact amount of the propelling powers of the peristaltic action, had to contend with considerable difficulties, the want of a proper apparatus, and its fastening within, or to the intestine, being the chief ones.

A glass tube was bent in appropriate angles and intimately connected with two small funnels, one to surround hermetically the superior fistula, and the other to be used as a recipient of water or any other fluid. This apparatus being applied and filled with cold water, produced at first contraction and firm closure of the opening; but after a while it relaxed, when the water descended, and disappeared within the fistula. The repeated contractions and relaxations of the entrail, thus raised and lowered the column of water in the glass tube. Gradually more water was added, so as to form a column 24″ in height. The pressure of the intestine upon its contents was amply sufficient to counterbalance it, (that is, a vertical column of water 24″ high,) nay, raising it even beyond its original level, and conjointly forcing out some alimentary contents.

Much beyond a column of water of the stated height the intestine could not balance, and though it acted with great vehemence, yet it could not prevent the water escaping into the artificial anus.

This experiment, however inefficient, tends nevertheless to prove:

1st. That during the peristaltic action, the walls of the intestines come in so close contact as to prevent the entrance of water.

2d. That the strength of the peristaltic action is amply proportioned to the pressure of a column of water 24 inches in height.

Again, a strong glass tube closed on one hand, and filled with twelve ounces of metallic quicksilver, was inserted in an acute angle to the horizon, and likewise propelled with great ease. Still other experiments were contemplated to measure the peristaltic force more accurately, but the patient objecting, they had to be abandoned.

### 3. *Succus Entericus.*

Its quantity is yet in dispute. Frerichs separated a portion of small intestine by ligatnre, and after a while it was filled with alkaline liquid. Bidder and Schmidt, however, state that the enteric juice just suffices to moisten the surface. The observations of Prof. Busch confirm the latter. By introducing a two-leafed speculum into the lower opening, a part of the mucous membrane became thus exposed. The surface, either pale or rose-colored, presented at no time an abundance of secretion ; nay, there was mostly not sufficient to moisten a stripe of litmus paper, if not left for a great while. Again, cane sugar, brought into the lower opening in a gauze bag, was, after 15 minutes exposure to the action of the enteric juice, not entirely dissolved. Hence it seems that this digestive fluid under physiological conditions is secreted, but in moderate quantity, and that its averted abundance is attributable either to bile, gastric, and pancreatic juices admixed, or to a morbid state. Mechanical irritation of the mucous membrane repeatedly augmented and deteriorated the enteric juice, converting it into a thick viscid substance not unlike the nasal mucus. The fluctuating consistency of enteric juice justifies the supposition that there is a difference in the proportion of fixed and liquid constituents. Bidder and Schmidt estimate the former at 1½ per cent. Busch has observed them to be as high as 7.04, and as low as 3.87, therefore, an average of 5.47 solids.

Chemical reaction of enteric juice was throughout alkaline.

### 4. *Digestive Properties of Succus Entericus.*

On this head the views of physiologists are almost diametrically opposed to each other. Frerichs has acquainted us with the power of this fluid to convert amylum into grape sugar, but disputes its effects upon protean substances, as asserted by Bidder and Schmidt. None of them had as good a chance in deciding the pending question more conclusively than Prof. Busch, on account of having enteric juice unadulterated for his observations.

As the first experiment may be admitted, the feeding of the patient, through the lower opening, with a diversity of nutriment, (beef tea, beer, farina soups, hard boiled eggs, and meat.) During six weeks prior to her entering the hospital, the patient had had but one alvine evacuation of the size of a chesnut, consisting probably but of mucous and epithelial cells. Subsequently she had every 24 hours a copious one of ordinary consistence though of a grayish white color, on account of the total absence of bile. Most conspicuous was their fœtid odor. Digestion seemed, otherwise, to be perfect, and the fæces retained no traces of nutriments taken. These facts, with the steady improvement of the patient, would seem to demonstrate the dissolving properties of enteric juice upon protein bodies.

In order to test separately the action of succus entericus upon various articles of food, they were introduced singly by means of permeable bags, whilst their respective weights were ascertained by the method of Bidder and Schmidt—that is, they were dried by 100° C., and their liquid parts evaporated prior to their insertion. After having remained in the bowel for some time, they were carefully washed with distilled water, again reduced to a dry state by evaporation, and the difference of weight noted. This method gives as near as possible an accurate result; inevitable accidental losses of food may be supposed compensated by retaining some of the mucous or enteric juice, notwithstanding the subjects of experiment being washed.

### 5. *Action of Enteric Juice upon Protein Substances.*

All experiments instituted for the purpose of ascertaining the solving faculties of enteric juice upon protein, resulted in a positive reduction and disintegration of the test substance. Although the loss of weight was but trifling, yet that is accounted for in the first place by the test substance being retained at a certain distance from the fistulous opening, where but a small quantity of the secreted juice could act upon it, and moreover that itself was freed from water, and hence in a very concentrated state.

When the test substances were removed from the bowel, they had lost their firmness, form, and color. The cubical pieces of egg had lost their corners and appeared like cheese; they crumbled in small fragments. Meat had become quite pale, and parted in rags and fibres. At the same time all presented evidences of advanced decomposition; a fetid odor and the contact with muriatic acid would cause the rise of ammonia vapors. That the latter originated in the putrescence of the nitrogenic articles, could not be doubted, for empty bags, or one filled with powdered charcoal, and for some time inserted into the bowel, would present no similar phenomena. Nor can it be said that the exposure of organic bodies to moisture and warm temperature would have produced those marks, as none of the experiments extended over 7 hours, but some were limited to 1½.

1. Experiment with Albumen.—1901 grains of hard-boiled albumen in a clean gauze bag, being introduced 6½ hours, then removed, washed and dried. It weighed 189 grains. 5589 grains of the same substance was also dried in the same manner, weighed 859 grains. According to that proportion, 189 grs. dry, represents 1229 grs. wet, leaving a balance of 672 grs. consumed by enteric digestion. Loss, 35.035 per cent.

2. Experiment with Albumen.—2298 grains hard-boiled albumen inserted for 5½ hours, afterward washed and dried, when it weighed 312 grs. For comparison, 4194 grs. of same egg being dried, showed a remnant of 0,609 grs.; consequently, 312 grs. dry, are equal to 2148 grs. undried albumen, showing a loss of 105 grs. or 6.05 per cent. by digestion.

Similar differences are noticed by Bidder and Schmidt.

1st Experiment with Meat.—On the 15th of January. 1605 grs. of boiled beef, in cubic pieces, were brought into the lower fistula. After 7 hours exposure to the enteric juice, it was removed and dried, when it weighed 0,528 grs. 4895 grs. of the same meat are equal to 2297 grs. when exsicated. In that proportion, 0,528 grs. dry, are equal to 1125 grs. of undried meat, and the loss sustained by digestion is, therefore, 0,480 grs., or 29.090 per cent.

2d Experiment with Meat.—3754 grs. boiled beef was subjected to the same process, and after 5 hours, its weight was ascertained to be 1,479 grs. dry. In a balancing experiment, 5094 grs. of same meat dried and reduced by evaporation to 2,124 grs.; a proportion from 3547 grs. to 1479, being equal to a loss of 5.03 per cent. by digestion.

### 6. *Action of Enteric Juice upon Starch.*

The enteric secretion acts by far more powerfully upon amylaceous substances, for the lowest per centage of loss by enteric digestion considerably exceeds the loss of protein, although variations were equally observed. In order to secure as correct a result as possible, Prof. Busch inserted the dried paste.

1st Experiment.—1286 grs. dried pap in a gauze bag, inserted for 5½ hours. When removed and perfectly dried, it weighed 0,469 grs., leaving a loss of 0,817 grs. by digestion.

2d Experiment.—1404 grs. dried paste, after 6 hours exposure, leave 0,863 grs.: a loss of 0,541 grs. or 38.5 per cent.

3d Experiment.—A glass with starch water was injected, and after 48 hours an injection was made of sulphate of magnesia, producing two stools; chemico-microscopical examinations of the latter show the perfect absence of amylum bodies. Whereas iodine solves them first red, gradually changing into pale violet. In all probability the fæces contained some dextrin, though no reduction of oxyd of copper could be effected.

The solution of starch in the intestine is attained by its being converted into grape sugar, and experiments to that effect showed invariably positive results under Fehling's test.

### 7. *Action upon Cane Sugar.*

Enteric juice does not change cane sugar.

1st Experiment.—A bag with small pieces of cane sugar was suspended in the lower opening; 17 minutes after they were not quite dissolved. The test gives no evidence of grape sugar, but sulphuric acid chemically verifies cane sugar.

2d Experiment.—Cane sugar was suspended for some hours in the bowel; the bag when removed contained no more pieces of sugar. Test, no grape, but cane sugar.

3d Experiment.—The bowels were cleared by a solution of Epsom salts. In proper time a solution of ℥ ii. of sugar is injected, and ℥ iss. is added in 10 hours. Shortly before the latter injection, patient had a serous stool, and soon after, a second; chemical inspection of both, slightly sour, no grape, but so much cane sugar was found, that after the liquid had been reduced by evaporation, a drop running down the wall of the vessel would have crystals along its course. The quantitative analysis yielded more than ℥ i. of cane sugar.

4th Experiment.—A very weak solution of sugar ℥ ii. to 600 cm. water was injected at 4 different times. Before, the stool was carefully examined and no sugar detected. after the second injection, a stool, in which was found, by careful proceeding, 5 grs. of cane sugar, but no grape sugar.

Prof. Busch is inclined to believe that the previous administration of sulphate of magnesia may have had some prejudicial influence upon the absorbents, yet his experiments clearly demonstrate that cane sugar is not converted into grape sugar by means of enteric juice. It should also be mentioned, that the chemico-microscopical analysis of the urine gave no evidence of either kind of sugar.

### 8. *Action of Enteric Juice upon Fat.*

There being no bile or pancreatic juice in the lower section of the intestinal track, it was a matter of great interest to ascertain what changes, if any, fat would undergo by the mere contact with enteric juice. For this special purpose Prof. Busch instituted the following experiment: During 10 days, the patient received small quantities of melted butter, in all ℥ iii. Unfortunately the anti-peristaltic motion ejected, at different times, small whitish-gray and sausage-formed substance, that proved to be fat, mechanically mixed with mucus and epithelium, *without forming* an intimate emulsion. The quantity thus eliminated from the fistulous opening could not be fixed.

On the 10th day, the patient had the first spontaneous discharge from the rectum, being whitish, and of a highly offensive odor. When cold it seemed to be enveloped with a hard fatty substance like tallow. The rest presented itself under the microscope as consisting of amorphous and crystallized fat, freely mixed with epithelium; the entire mass reacted sour, and the smell manifested rancid acid. By repeatedly shaking the fæces with sulphuric ether, and subsequent evaporation, about ℥ ss. of fat could be collected.

In another experiment, ℥ iv. of oleum jecoris Aselli were injected at intermissions. A good deal was lost by anti-peristaltic motion. The small evacuation that ensued 14 days after consisted of large fat drops with mucus and epithelium. About the fæces there was more than an ounce of liquid oil.

### *Observations upon the Superior Portion of the Alimentary Track.*

It will be remembered that the left fistulous opening terminated the superior section of the intestinal track at about the beginning of the jejunum. Before breakfast, synchronistic with the peristaltic motion, a discharge of digestive fluids was observed that was rendered frothy by the atmospheric air in the intestine.

The statements of Beaumont and other observers, indicate that food requires some considerable time before it leaves the stomach. Prof. Busch says, however, meat, eggs and vegetables appear at the opening from 15 to 30 minutes after being taken. From a large number of observations on this point, he relates, that

| | | | | |
|---|---|---|---|---|
| Boiled | eggs | were discharged in | 20, 26, 35 | minutes. |
| " | cabbage | " | 15 to 19 | " |
| " | meat | " | 22 to 30 | " |
| " | carrots | " | 12 | " |
| " | potatoes | " | 15 | " |

A sumptuous meal required from 3 to 4 hours for its entire removal; but traces could be detected even beyond that time, and among nourishments subsequently consumed.

With evening meals it was different; but a part was discharged, and the rest next morning.

The chemical reaction of digestive fluids in the morning was always neutral; the enteric juice, however, alkaline. When mixed with food, the reaction differed so much as not to elicit any stationary rule. Although solids appeared to the eye unaltered, yet a mere touch indicated their disintegration and softening. As stated by others, the microscope revealed the muscular fibres being split lengthways and broken across; and in a greater measure, when the meat had previously been hashed so as to expose more surface.

Mixed food was always discharged with a large quantity of digestive fluids, so as to swim in it; uniform aliments and some vegetables, cabbage, turnips, carrots, and potatoes, diminished the latter considerably. Amylaceous substances were but in part converted into grape sugar; protein ones gave ordinarily no evidence of albumen, from which, however, two slight exceptions were noticed.

The digestive fluids were so intimately mixed as to render examination almost useless. But one point was decided, namely, the complete absence of saliva by the negative result upon rhodanpotassium, caused either by previous resorption or chemical appropriation. The considerable quantity of digestive fluids discharged in the morning justified the supposition, that the gastric juice supplied a large proportion, which did, however, not conform with the average amount of ashes by dry distillation, namely, 2.048 per cent., whilst gastric juice is said to furnish 3 per cent. solids.

The number of heterologous substances entering upon the composition of ordinary chyme would render any examination doubtful in result, hence the experiments had to be directed upon simple material.

### 1. *Cane Sugar.*

The action of gastric juice upon cane sugar is yet a question of dispute. Bouchardat contends that sugar is first to be converted into grape sugar before it is capable of being absorbed ; this conversion he ascribes to the effects of gastric juice, whereas Frerichs believes that the simple solution of cane sugar is directly absorbed by the stomach.

In order to render the experiments conclusive, the patient was permitted to take no other food, on the previous day, than meat and eggs. The digestive fluids, despite the presence of bile, presented next morning, *no evidence of grape sugar.* After this negative fact had been ascertained, 2 ounces of cane sugar were dissolved in 28 ounces of water, and gradually taken by the patient. Under the same precautious ℥iii. of cane sugar, dissolved in a large quantity of water, were at another time administered to the patient. In both instances but grape sugar was discharged from the fistulous opening, and no trace of cane sugar; proving conclusively the entire conversion of the latter into grape sugar.

2. *Fluid Albumen.*

With a view of testing the coagulability of albumen by gastric juice, being still asserted by some physiologists, the patient received the albuminous portion of four eggs, diluted in water. During the following four hours a moderate quantity of an opaque, sticky, and alkaline fluid was discharged and collected, in which no fragment of coagulated albumen could be noticed. Part of that fluid, diluted with distilled water, was subjected to the following tests:

(*a.*) *Boiling* produces coagulation.

(*b.*) *Alcohol* precipitates numerous flakes.

(*c.*) *Nitric acid* in minimo: coagulation; being boiled, perfect solution; refrigerated, precipitates again; acid added and boiled, dissolves and remains so.

(*d.*) *Acetic acid* in minimo: no effect; boiled, coagulates; acid added, dissolves the precipitate; liquid cold, opalesces; becomes clear again on boiling.

The same results were attained by the same tests upon fresh albumen.

From the preceding, it follows that albumen experiences no change by the stomach.

How much of it is absorbed by this organ could not be ascertained with any degree of certainty, on account of its being intimately mixed with mucus, bile, &c. However, some experiments were made to that effect.

In one instance the patient, having received on the evening previous *but* vegetable food, took 38 drachms raw albumen. In order to control the experiment, 349 grains of raw albumen were dried, the water free remnant amounting to 2,708 grains. The discharged fluid being collected, acidulated and boiled; the precipitate washed with water, alcohol, and ether, in order to free it as much as possible of choleviridine, bile, fat, and other foreign substance, finally dried, leaves 6,137 grains solids. In proportion with the former ʒ xxii had been absorbed, and the rest passed into the small intestine.

### 3. *Gummi.*

One morning the patient took ℥ i. gum arabic with water. Unfortunately its entire elimination could not be waited for, because the patient was so much tortured by hunger as to demand satiation after two hours. During this time 142 cm. of a sticky, green, and neutral substance had run out, in which there was *no sugar*. 15 cm. of it were diluted with water, by which it became almost transparent. With alcohol it forms a considerable white and thick precipitate. The latter being filtered and warmed with water, dissolves again, leaving but a few particles (mucus.) The quantity of water being increased, all filtered, and in fine evaporated, the solids weigh 2,126 grs., according to which, 142 cm. contain 20,126 grains of gum. It follows, that within the short period of two hours, about two-thirds of gum consumed had again been discharged.

In another experiment, patient had taken along with soup in the morning and noon, a considerable quantity of gum arabic. The same process was gone through as in the former experiment, and its result was materially confirmative.

### 4. *Gelatina.*

Before breakfast, ℥ v. of gelatina, with an addition of wine to render it palatable. Within 3½ hours a sticky, neutral fluid issued from the aperture. It was carefully examined, and found that two-thirds had been absorbed.

### 5. *Milk.*

After taking milk, a sour liquid was collected, in which small crumbs, and not large pieces of casein, as seen in children, were swimming. Being filtered, acetic acid caused some coagulation, soluble on adding acid. In that solution, cyanoretum ferro-potassii effected opalescence, nitric, muriatic, and sulphuric acids, bi-chloride of mercury, alum, and alcohol, produce precipitation, which precipitate would dissolve again by adding more test substance.

Examining upon sugar—positive result.

Evidently had not all casein coagulated, and in part remained solved in the fluid.

### 6. *Fat.*

In the morning cod-liver oil; discharge of a large quantity of fluid ensued, disproportionate with the amount of oil taken.

1st. Experiment.—Ol. jecoris Aselli ʒ xiv., within three hours almost ℥ x. of dark gray fluid escaped.

2d Experiment.—Ol. jecoris Aselli ʒ xv., in same time ℥ xii. ʒ vi. fluid run out.

Reaction different, mostly sour, yet occasionally alkaline; in the latter case the fat was so finely diffused as not to be visible to the naked eye. Under the microscope, however, it appears as infinite small fat molecules. The liquid, when sour, represented as near as possible a perfect emulsion, yet some oil drops would swim on its surface. If the primarily alkaline liquid, by exposure to air and temperature, had subseqently turned sour, a part of the fat would separate and swim on the surface, as before mentioned.

### *Digestibility of Different Aliments.*

To determine the digestibility of food is of so great and practical interest, that various means have been devised and employed. The case of Busch seemed to be particularly calculated of serving for such experiments.

With reference to sugar, it has already been stated that in one instance (℥ ii.) the thirtieth, and in another (℥ iii.) the fifteenth part had entered the small intestines, whilst the rest had been appropriated on its way towards the fistulous aperture. As to fluid albumen, the proportions absorbed to those appearing in the jejunum are 5, 6 : 3, 2, and to gelatine about 2 : 1.

The plan adopted by Prof. Busch, of ascertaining as near as possible the relative digestibility of consumptibles was:

(*a.*) That in the morning a certain quantity of a certain aliment was given to the patient.

(*b.*) That the solids of that aliment were exactly made out by drying.

(*c.*) That the fluid discharged thereupon was collected until the fragments of that aliment disappeared, then weighed, filtered, and reduced to dry state by evaporation.

(*d.*) That its relative proportions were ascertained arithmetically.

As a matter of course, those calculations are not exact, because it is impossible to fix the amount of digestiue fluid partaking in the formation of chyme, though it was duly taken in consideration that certain alimentary articles employed a larger proportion of digestive fluids than others.

1st Experiment.—Patient takes thirty-nine drachms of soft-boiled eggs; 3,975 grs. contain 985 grs. solids, equal to 24.78 per cent.; the patient has consequently received 585 grs. of solids. During the experiment no drinks permitted. After four hours the evidence of albumen disappeared; the entire liquid collected within this time amounts to ℥ xiii, 150 grs.; part of the fluid well stirred and dried, leave but 6.86 per cent.; another well filtered and dried, leave but 4.8 per cent.

2d Experiment.—℥ iv., ʒ vss. of hashed roast veal eaten by the patient; 6,415 grs. when dried leave 2,466 grs. or 38.4 per cent. solids, equal to ℥ j., ʒ iv., grs. 50 of solids in the veal consumed. After three hours and forty minutes,

℥ viij., ʒ j. of fluid have been collected, when no further traces of meat could be noticed. Part of the liquid being filtered and dried, it shows 5.4 per cent. solids, the non-filtered leaves 7.78 per cent.

3d. Experiment.—℥ xvii., ʒ vi. milk are drank; 26.105 grs. dried give 8 19 per cent.; in all there are 2,907 solids. In four and a half hours ℥ xxii., ʒ ii. of a sour fluid have been collected, which being dried, the filtered furnished 3.4 per cent., the non-filtered 4.08 per cent.

4th Experiment.—℥ xxx., ℥ vj. cabbage, cooked with fat, eaten; 15,596 grs. of it leave 1,586 grs. or 10.2 per cent., equal to ℥ iij., ʒ j. solids. The first pieces of cabbage discharged were almost unaltered, and but comparatiuely little fluid could be collected during the subsequent four hours. The latter, treated with distilled water, and well filtered, furnished 2.64 per cent. solids, the non-filtered 6.6 per cent.

5th Experiment.—℔ ii., ℥ ix., ʒ iii. boiled carrots, eaten; 17,646 grs. contain 2,818 grs. or 1,597 solids. After four and a half hours, ℔ iij., ℥ ij., ʒ vi. of a consistent pap is discharged; 17,985 grs. being filtered and dried, return 0,560 grs. or 3.1 per cent. solids in solution; the rest being subject to same process without filtration, return 6.4 per cent.

6th Experiment.—℔ ij., ℥ xii., ʒ ij. potato pap, prepared with broth and some fat. In 12,986 grs. of this food, 2,926 grs. or 22.5 per cent. are solids. After four and a half hours, but mere traces of potato can be observed. During that time ℔ ij. ʒ vi. of a thin saccharine pap has been collected; 24,029 grs. filtered and washed. In a soluble state, 1,548 grs. or 6.4 per cent. of solids are found; 21.465 grs. are dried without filtration, and 10.6 per cent. are found.

The result of these and other experiments elicit:

| | Proportionate weight between Aliment taken and Chyme discharged. | Proportionate weight of Solids taken, to the Solids in Chyme. | Proportionate weight of solved, to insolved Solids in Chyme. |
|---|---|---|---|
| Fat . . . | 1.6,0 | | |
| Gelatine . . | 1.3,675 | 1.0,94 | |
| Boiled eggs . | 1.2,73 | 1.0,76 | 1.2,3 |
| Meat . . . | 1.1,73 | 1.0,35 | 1.2,27 |
| Milk . . . | 1.1,25 | 1.0,62 | 1.4,3 |
| Carrots . . | 1.1,2 | 1.0,49 | 1.0,94 |
| Cabbage . . | 1.0,91 | 1.0,58 | 1.0,66 |
| Potato . . | 1.0,7 | 1.0,53 | 1.1,5 |

The first column of the preceding table demonstrates clearly the quantitative differences of digestive fluid required by different alimentary material. Oleous and protein substances employ by far a larger quantity than vegetables.

The chyme being made poorer in solids than the aliments taken, is consequently a higher dilution with water, the loss of which the patient had to replace by frequent drinks.

The solids introduced exceed considerably the solids discharged, despite the profuse admixture of digestive fluids; yet the comparison of the different figures in the several columns above could not be considered positive, without calculating also the quantity of digestive fluids employed, (vide gelatine.) But the conclusion seems to warrant, that meat is more digestible than eggs; carrots more than potatoes; and the latter more than cabbage.

With a view to ascertain the difference in the appropriation of alimentary material of purely animal or mixed diet, the following experiments were instituted:

Consumed in twenty-four hours, were—

| | | | |
|---|---|---|---|
| Beef tea, mixed with 3 eggs, . . | ℔ 2 | $24\frac{31}{32}$ | loth. |
| Two boiled eggs, . . . . . | 0 | $5\frac{5}{16}$ | " |
| Roast pork, . . . . . . . | 0 | $8\frac{1}{2}$ | " |
| Beef, . . . . . . . . | 0 | $17\frac{5}{16}$ | " |
| Smoked tongue and cold roast veal, . | 0 | $18\frac{1}{2}$ | " |
| Water, . . . . . . . . | 2 | $2\frac{1}{2}$ | " |
| | ℔ 6 | $12\frac{19}{32}$ | " |

| | | | |
|---|---|---|---|
| | ℔ 6 | $12\frac{19}{32}$ | loth.* |
| From the fistulous aperture discharge, | 5 | $9\frac{1}{4}$ | |
| Balance, . . . . . . . | 1 | $3\frac{11}{32}$ | " |
| Urine, . . . . . . . . | | $28\frac{27}{32}$ | " |
| Rest, . . . . . . . . | | $6\frac{1}{2}$ | " |

On another occasion—

| | | | |
|---|---|---|---|
| Coffee, . . . . . . . | ℔ 2 | 1 | loth. |
| Broth mixed with eggs, . . . | 2 | $29\frac{1}{2}$ | " |
| Bread (rye bread,) . . . . . | 0 | $16\frac{1}{4}$ | " |
| Rusk, (wheat bread,) . . . . | 0 | 8 | " |
| Meat, . . . . . . . . | 0 | 11 | " |
| Carrots and potatoes, . . . . | 1 | $5\frac{3}{4}$ | " |
| Soup (amylaceous,) . . . . . | 1 | 2 | " |
| Wine, . . . . . . . . | 0 | $15\frac{7}{8}$ | " |
| Water, . . . . . . . . | 1 | $17\frac{1}{2}$ | " |
| | 10 | $10\frac{7}{8}$ | |
| From the fistula discharge, . . . | 7 | $3\frac{3}{4}$ | " |
| Balance, . . . . . . . . | 3 | $7\frac{1}{8}$ | " |
| Urine secreted in same period, . . | | $14\frac{5}{16}$ | " |
| Remains, . . . . . . . . | 2 | $24\frac{13}{16}$ | " |

From the first experiment may be inferred that, after deducting the urinary secretion and the discharge from the fistulous opening from the weight of food and drinks received, no balance remained sufficient to sustain the other expenditure of the body through lungs and skin; whilst the second experiment left a sufficient balance for the purposes of the organism.

Most conspicuous were the changes in the chyme of the first experiment, when compared with the beginning and termination. The discharged chyme was warm examined every three hours. At first but a liquid run out, in which crumbs of egg, meat, &c., swam; gradually the amount

* 1 loth of German weight = ℥ iv.

of digestive fluid decreased in the same ratio as the solids increased, so that, particularly in the morning, a consistent pap eliminated from the opening, looking and smelling like fresh meat, without being even colored by bilicidrose. In the latter experiment the chyme remained fluid throughout, with an exception at noon, when it became for a short while more consistent.

### 8. *The Solving Power of Digestive Fluid.*

On filtering the chymes in which fragments of egg or meat were suspended, it was noticed that their solution proceeded in the open air without putrescence, and that but a small quantity remained undissolved upon the filterer. This took place, whether the digestive liquids were neutral or alkaline. All those protein substances having for a time been exposed to the exclusive action of gastric juice, it became a point of interest to learn how much of fish, egg or meat might be dissolved by the conjoint action of all digestive fluids; and for this purpose the following experiments were undertaken:

1st Experiment.—After the taking of raw albumen, the alkaline reacting liquid was collected, and 910 grs. of boiled albumen suspended in a gauze bag, part of the latter having previously been dried, and its solids fixed upon, 15.3 per cent. After six hours' exposure, the cubic-shaped pieces had lost their contours, and showed a tendency of dissolving in small particles and flakes. They had in weight, collectively, lost 41 grs. (This artificial digestion had been effected in low temperature, to avoid fermentation.)

2d Experiment.—The patient having previously consumed some meat, the liquid portion of discharged chyme, being neutral, was mixed with 1,648 grs. of cold roast veal, cut in square pieces, and placed for twenty hours in a cold room. At the termination of the experiment, no putrescence; the surface of the meat is very soft and imbued by biline liquid. The examination elicited a loss of 555 grs. of meat.

It is obvious that the neutral, and even alkaline mixture of digestive fluids, retain their digestive powers upon protein substances without the body, although not in the same degree; and the exterior juice being also endowed with digestive properties, it seems to be conclusive, that the gastric digestion is the prominent one, and the other merely supplementary.

### 9. *Quantity of Digestive Fluids.*

The patient received for 12 hours but $2\frac{1}{4}$ lbs. of black rye bread and water at pleasure. The first chyme was neutral; the latter sour. A good quantity of tolerably consistent bread chyme was discharged before the clear, digestive fluid appeared. The entire liquid collected during 24 hours amounted to 5 lb. $21\frac{1}{8}$ loth. After deducting the weight of the bread it would leave a fluid balance of 3 lb. $5\frac{1}{8}$ loth. Yet this calculation is considerably altered by the fact that the bread contained $42\frac{66}{100}$ loth, (21 oz.,) whilst the entire discharge contains but about 1 lb. of solids, including the uncountable solids of the digestive fluids. Dr. Busch assumes that about 4 lbs. of digestive fluids have been expended in the experiment, which was the seventeenth part of the entire weight of the body, (68 lbs.)

Prof. Busch sums up the results of his experiments as follows.

1. Hunger is constituted by two sensations; the first is represented by the nervous system in general, and derived from the impoverished condition of the tissues; the second originates with the nerves of the digestive organs, indicating their emptiness. The former is removed only by the required assimilation of nutritive elements, and not by merely filling the first passages.

2. The peristaltic motion of the intestines take place with the same power within the abdominal cavity as when exposed to the atmospheric air. Its propelling power equals a column of water 24 inches high.

3. The alimentary canal has its periods of rest and action.

4. The quantity of enteric juice secreted is invariably small, and of alkaline reaction. Its percentage of solids averages 5.47.

5. Enteric juice is capablo of digesting amylaceous and protein substances.

6. Enteric juice converts starch into grape sugar.

7. Enteric juice prepares protein substances for assimilation under the phenomena of putrescence.

8. Enteric juice leaves cane sugar unchanged.

9. Cane sugar, absorbed as such, is not discharged in the urine.

10. Fat, unless exposed to the action of bile or pancreatic juice, is absorbed either not at all or in insignificant quantity.

11. Food appears between 15 and 30 minutes after being taken in the superior third of the thin intestine.

12. Solution of cane sugar disappears in part before entering the small intestine: all that enters the latter is converted into grape sugar.

13. Raw albumen taken from hens' eggs is directly absorbed in the stomach and the adjoining portion of the small intestine. All that descends to the lower portion of the latter is unchanged.

14. Gum is not converted into sugar, but remains unchanged.

15. Gelatine is dissolved, and loses thereby its coagulability.

16. Casein remains partly dissolved in the digestive fluids.

17. Fat is entirely emulgated by the digestive fluids when alkaline or neutral, but partially when acid.

18. The digestive liquids of the small intestines possess digestive powers over protein substances.

10. The minimum of all digestive fluids entering the small intestine in the course of 24 hours, amounts to more than the seventeenth part of the weight of the body.

[*Am. Med. Gaz.*

